# Inebilizumab treatment in a patient with co-occurring AQP4-IgG positive neuromyelitis optica spectrum disorder and myasthenia gravis: a case report and literature review

**DOI:** 10.3389/fimmu.2024.1528989

**Published:** 2025-01-13

**Authors:** Xiaoqian Song, Jingjiao Chen, Chenyang Jin, Yilong Peng, Yuewen Sun, Xueping Zheng

**Affiliations:** ^1^ Department of Geriatric Medicine, Affiliated Hospital of Qingdao University, Qingdao, China; ^2^ Qingdao Medical College, Qingdao University, Qingdao, China

**Keywords:** neuromyelitis optica spectrum disorder, myasthenia gravis, inebilizumab, anti-AQP4 antibodies, anti-acetylcholine receptors antibodies

## Abstract

**Objective:**

This study aims to delineate the clinical features underlying the concurrent disease of neuromyelitis optica spectrum disorder (NMOSD) and myasthenia gravis (MG), and to identify efficacious therapeutic strategies.

**Background:**

NMOSD and MG are uncommon autoimmune diseases that infrequently co-exist. Despite previous reports, a consensus on treating NMOSD concurrent with MG is lacking.

**Methods:**

We present the case of a 55-year-old female with both anti-aquaporin-4 (AQP4) antibody-positive NMOSD and anti-acetylcholine receptor (AChR) antibody-positive MG, who achieved stable disease control following treatment with inebilizumab without significant adverse effects. We also conducted a literature review to evaluate the clinical profile of this comorbidity.

**Results:**

Our review identified 85 patients with concurrent NMOSD and MG. In 70 well-documented cases, MG predated NMOSD in 60 (85.8%) cases, with 42 (70%) patients having undergone thymectomy. Six (8.6%) patients were first diagnosed with NMOSD, and then thymectomy was performed in 2 (33.3%) MG patients. For NMOSD treatment, although most patients received steroid hormones and immunosuppressive agents, quite a few patients had persistent severe disability. Additionally, of 44 patients with clear records of disease recurrence, 31 patients(70.5%) experienced frequent relapses of optic neuritis and myelitis, ranging from 1 to 15 attacks, averaging five. The manifestations of MG are mainly included fatigability, diplopia, and blepharoptosis, with symptoms well-controlled in most patients. Our patient treated with inebilizumab for 1 year and no relapse was recorded to date.

**Conclusions:**

Though MG typically precedes NMOSD and thymectomy is frequently performed, it is not a prerequisite for NMOSD development but may represent a potential risk factor. MG generally follows a benign course, in contrast to the more aggressive nature of NMOSD. The utility of biological agents such as inebilizumab for patients with both NMOSD combined with MG warrants further attention.

## Introduction

Neuromyelitis optica spectrum disorder (NMOSD) is an uncommon demyelinating disorder of the central nervous system (CNS) caused by the action of the anti-AQP4 antibodies (1). It is marked by recurrent episodes of optic neuritis(ON) and myelitis, which often lead to significant disability if not treated promptly. Myasthenia gravis(MG) is a chronic organ-specific autoimmune disorder caused by antibody-mediated attacks on nicotinic acetylcholine receptors(AChR) at the neuromuscular junction. This interference disrupts neuromuscular transmission, ultimately leading to muscle weakness and fatigue (2). Moreover, the prevalence of MG varies between 0.8 and 20 per 100,000 population (3), and prevalence of NMOSD is reported as ranged from 0.07 to 10 per 100,000 (4). Although both disorders are rare, the coexistence of MG and NMOSD occurs much more common than by chance, with over 100 cases reported to date (5). And a study reported five of 214 reviewed patients with MG (2.3%) who had CNS demyelinating lesion or disease (6).

This co-occurrence can complicate the diagnosis of both conditions, as overlapping symptoms may obscure clinical presentation. Furthermore, the concurrent presence of MG complicates NMOSD treatment and may contribute to poorer outcomes (7). In this report, we described a patient with concurrent NMOSD and MG, highlighting their clinical course and the challenges encountered in managing these overlapping autoimmune disorders. In addition, we provide a comprehensive review of similar cases in the literature to better delineate the clinical features, potential pathogenic mechanisms, and effective therapeutic strategies for patients with these coexisting conditions. Understanding the interplay between NMOSD and MG is crucial for optimizing management, improving outcomes, and guiding future research into these complex autoimmune diseases.

## Case report

A 55-year-old female was hospitalized in May 2023 due to dizziness, nausea, vomiting and gait disturbance. She had been diagnosed with MG over 10 years and had received intermittent treatment with traditional Chinese medicine. Ten years ago, she experienced episodes of ptosis and dysarthria, which was improved completely with traditional Chinese medicine. Upon this admission, she exhibited no MG symptoms, such as ptosis, dysarthria, or fatigue.

**Figure 1:**
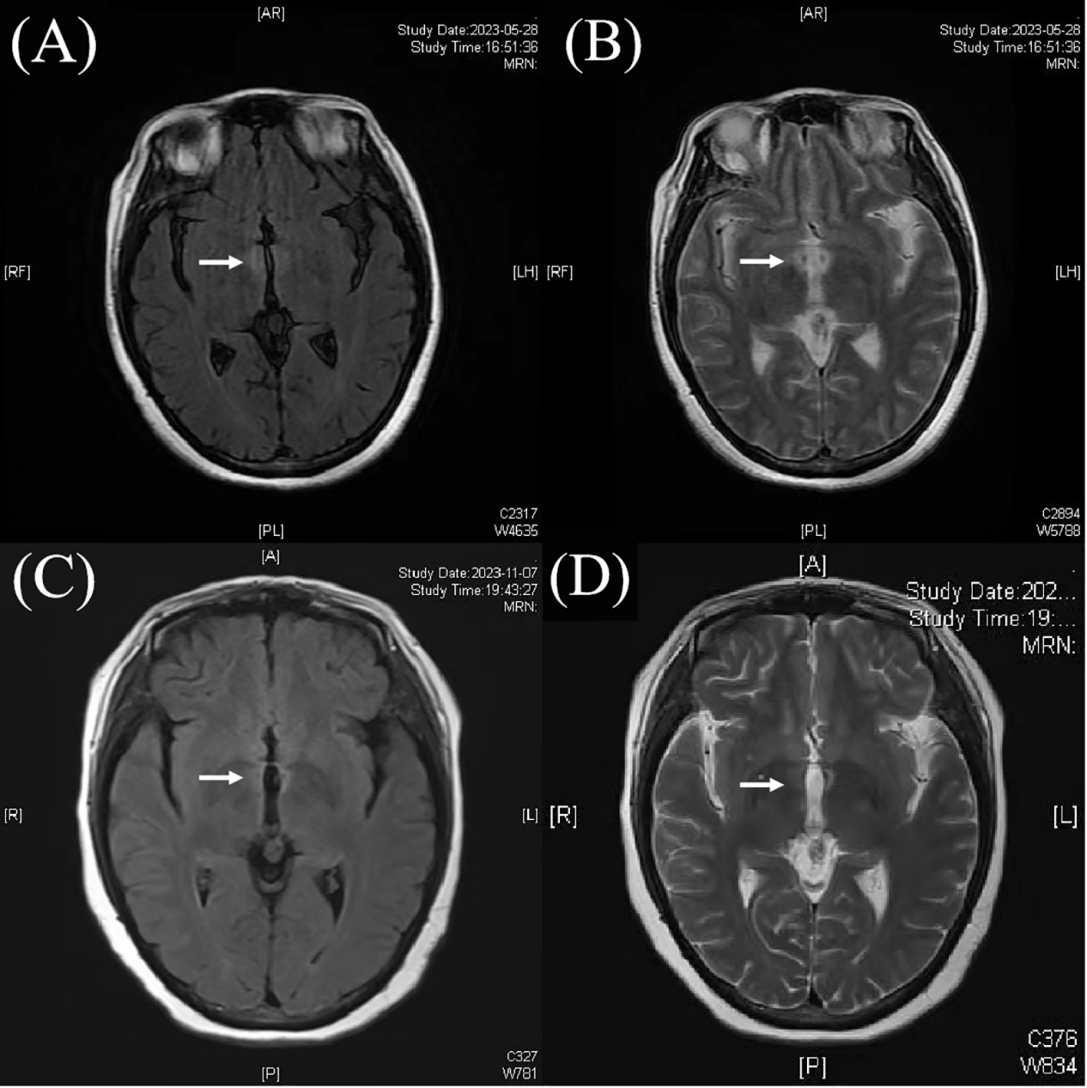
Magnetic resonance images (MRI) showed abnormal lesions around the third ventricle **(A, B)**. After treatment, the abnormal lesions of the third ventricle was weaker than before **(C, D)**.

Brain magnetic resonance images (MRI) revealed abnormal lesions around the third ventricle (**Figure 1**). Wernicke’s encephalopathy was first considered due to the brain MRI lesions, but was then ruled out because of her persistent symptoms without improvement. Cerebrospinal fluid (CSF) examination demonstrated a normal protein level (404mg/L, normal range 120-600mg/L) and white blood cell count of 8/mm^3^(normal range 0-8/mm^3^). Thyroid testing indicated anti-thyroglobulin antibody level was 669 IU/mL (normal range <115 IU/ml), and anti-thyroid peroxidase antibody level was 170 IU/mL (normal range <34 IU/ml). Thyroid ultrasound findings were consistent with Hashimoto’s thyroiditis. Serum analysis showed a positive AQP4 antibody titer of 1:1000(cell-based assay). Given the high specificity of AQP4 antibodies, she was diagnosed with NMOSD. Then she received intravenous methylprednisolone (500 mg/day) for 5 days, which partially improved her neurological symptoms. She was discharged on oral prednisone (50 mg/day) and mycophenolate mofetil (MMF) (500 mg twice daily). Five months later, she presented with visual blurring in right eye with visual acuity reduced to 0.12 over about 2 weeks. At this admission, her visual evoked potential response was absent on the right eye. Neurological examination showed grade V muscle strength in all limbs. Her quantitative myasthenia gravis score (QMGS) was 0 and Expanded Disability Status Scale (EDSS) score was 3. Her anti-AChR antibodies were positive (6.36 nmol/L, serum ELISA, normal range <0.45nmol/L),while other autoantibodies including MuSK, LRP4, RyR, Titin, antinuclear antibodies (ANA) profile, anti-neutrophil cytoplasmic antibodies (ANCA), and T-SPOT testing were negative. Chest CT showed no thymus abnormalities, and spinal and orbital MRI revealed no notable findings.

She was then treated with methylprednisolone (1 g/day for 5 days) and initiated with inebilizumab (300 mg at day 0,15, and then every 6 months). After two doses, her CD19 B-cell count decreased to 0.1%, and she tolerated well without adverse effects. Over the following year, the patient remained relapse-free after 3 doses, with a stable QMGS of 0 and EDSS score of 2.

## Results: literature review and our case

A total of 27 articles were searched, including a cohort study, a multicentre study (8, 9). Those cases were identified with a PubMed search using the terms “neuromyelitis optica spectrum disorder,” “neuromyelitis optica,” “myelitis,” “optic neuritis,” “multiple sclerosis” and “myasthenia gravis.”

A total of 85 patients were identified with comorbid NMOSD and MG, including our patient (**Table 1**). Among 70 patients with well-documented reports, MG predated NMOSD in 60 (85.8%) cases, with 54 (94.7%) being female. MG onset occurred between the ages of 8 and 63 years (median 27.5 years). Of these, 46 patients (76.7%) were aged ≤50 years. Forty-two (70%) had undergone thymectomy, with thymoma confirmed in 3 cases. NMOSD occurred first in 6 cases, with 5 patients (83.3%) being female, and one patient (16.7%) being male. NMOSD onset occurred between the ages of 16 and 79 years (median 41.5 years). Two of these patients had undergone thymectomy, one confirmed case of thymoma. Among patients with both MG and NMOSD, 74 patients (91.3%) were positive for anti-AChR antibodies, and anti-AQP4 antibodies were detected in 62 cases (83.8%). Thirteen patients (26%) also had concurrent other autoimmune diseases, such as thyroid disease, systemic lupus erythematosus (SLE), and Sjögren’s syndrome (SS). Additionally, 9 patients (18%) tested positive for other immune-related antibodies, including ANA and double-stranded DNA (dsDNA). These results are summarized in **Table 1**.

**Table 1:** Clinical and laboratory findings in 50 patients with MG and NMOSD.

No	Sex	Age at onset MG	Age at thymectomy	Histology of thymus	Age at onset of NMOSD	MG treatment	MG prognosis	First Attack of NMOSD	Symptoms at NMOSD onset	No. of ON attacks	No. of Myelitis attacks	NMOSD Treatment (acute phases)	NMOSD Treatment (remission phases)	NMOSD prognosis	Anti-AchR-Ab	Anti-AQP-4-IgG	Other immunological diseases or related antibodies
Uzawa (2)	F	20	28	hyperplasia	41	thymectomy, prednisolone, PE, IVMP, tacrolimus	ptosis and nasal voice were occasionally observed	ON	acute visual loss	4	4	IVMP,PE	prednisolone	severe disabilities persisted	P	P	NA
Uzawa (2)	F	18	20	NA	29	steroid, thymectomy	clinical remission	ON	NA	8	3	IVMP	prednisolone	severe tetraplegia and visual loss persisted	N	P	NA
Isbister (10)	F	28	NA	hyperplasia	36	NA	NA	ON	NA	NA	NA	NA	NA	NA	P	NA	NA
Kay (11)	F	44	not done	not done	49	pyridostigmine	remission	NMOSD	weakness, abnormal sensibility in all limbs, urinary and fecal incontinence	3	3	prednisone AZA	prednisone, AZA	marked and prompt improvement of symptoms.	P	P	NA
Furukawa (12)	F	23	NA	hyperplasia	48	thymectomy	clinical remission	NMOSD	band-like thoracic pain, ophthalmic pain	1	1	IVMP	prednisolone	symptoms improved modestly	P (1304)	N	chronic thyroiditis, ANA 1: 720
Kister (13)	F	38	38	hyperplasia	39	pyridostigmine bromide, thymectomy	symptoms resolved	ON	acute visual loss	7	3	AZA	NA	She had only shade perception and ambulated with assistance	P	P	stiff-person syndrome.
Kister (13)	F	36	37	hyperplasia	42	pyridostigmine, thymectomy	symptoms resolved	ON	acute visual loss	1	2	IVMP, IVIG	methylprednisolone AZA	NA	P	P	ANA1:80 Anti-GAD
Kister (13)	F	17	17	hyperplasia	19	pyrostigmine, thymectomy	well-controlled	ON	acute visual loss	6	9	NA	NA	required a cane for walking	P	N	ANA1:1024, anti-cardiolipin antibodies
Kister (13)	F	27	28	NA	38	pyridostigmine, prednisone, thymectomy, AZA	clinical remission	NMOSD	vision acutely declined, weakness, abnormal sensation and urinary retention	2	2	NA	NA	vision deteriorated acutely in both eyes	P	NA	NA
Nakamura (14)	F	28	30	NA	38	thymectomy	NA	ON	NA	7	2	IVMP	methylprednisolone	sensory disturbance	P	P	NA
Bichuetti (15)	F	26	27	hyperplasia	32	pyridostigmine, thymectomy	NA	NMOSD	truncal and gait ataxia associated to horizontal nystagmus	NA	NA	IVMP	methylprednisolone	symptoms resolved	NA	P	NA
Bichuetti (15)	M	27	not done	not done	45	pyridostigmine, AZA, cyclosporine	remain stable	ON	pain, low visual acuity	NA	NA	NA	cyclosporine	symptoms resolved	NA	P	NA
Bichuetti (15)	F	27	28	NA	31	prednisone, thymectomy, pyridostigmine, AZA	NA	AM	left side paresthesia	3	2	IVMP	prednisone AZA	symptoms resolved	NA	NA	ANA 1:320
Kohsaka (16)	F	NA	NA	hyperplasia	60	cortin, thymectomy, cyclosporine	NA	AM	NA	NA	NA	IVMP , tacrolimus	NA	NA	P	NA	NA
Ogaki (17)	F	30	40	NA	43	pyridostigmine bromide, thymectomy	well-controlled	ON	visual field defect in left eye	2	2	prednisone	metacortandracin	NA	P	P	ANA1:1280, hyperthyroidism
Hironishi (18)	F	23	NA	NA	30	thymectomy	complete remission	MNOSD	NA	NA	NA	IVMP	steroid AZA	NA	P	NA	NA
Jarius (19)	F	31	36	hyperplasia	59	thymectomy	NA	AM	NA	0	2	NA	NA	NA	P (6.8)	P	ANA 1:640, TPO-Ab
Jarius (19)	F	26	not done	not done	26	NA	NA	ON	NA	several	2	NA	NA	NA	P (26.3)	P	ANA
Jarius (19)	F	23	23	hyperplasia	43	NA	NA	AM	NA	0	3	NA	NA	NA	P (29)	P	ANA, anti-dsDNA-Ab
Jarius (19)	F	25	not done	not done	66	NA	NA	AM	NA	0	2	NA	NA	NA	NA	P	ANA
Jarius (19)	F	21	35	hyperplasia	67	NA	NA	AM	NA	0	2	NA	NA	NA	P (12.5)	P	anti- GAD Ab,
Jarius (19)	F	33	35	normal	47	NA	NA	AM	NA	3	2	NA	NA	NA	P	P	SS
Jarius (19)	F	13	14	normal	28	NA	NA	ON	NA	1	1	NA	NA	NA	P	P	NA
Jarius (19)	F	11	13	thymitis	32	NA	NA	ON	NA	1	1	NA	NA	NA	P (63.4)	P	SLE
Jarius (19)	F	29	not done	not done	55	NA	NA	ON	NA	1	2	NA	NA	NA	P (7.6)	P	NA
Spillane (20)	F	23	24	normal	31	pyridostigmine, prednisolone, thymectomy	remain stable	MNOSD	nausea, vomiting and hiccups, altered sensation in right arm	0	1	prednisolone, AZA	NA	limb power returned to normal	P	P	NA
Ikeguchi (21)	F	25	29	normal	49	prednisolone, thymectomy	symptoms improved	AM	gait disturbance	0	1	IVMP	prednisolone	able to walk unassisted	P	P	NA
Ikeguchi (21)	F	47	not done	not done	79	prednisolone	NA	ON	acute vision loss in left eye	2	0	IVMP	prednisolone	right eye blindness and disability	P	P	NA
Ikeguchi (21)	M	41 (preceded NMOSD)	41	thymoma	41	steroid	NA	NMOSD	NA	NA	0	steroid	NA	NA	P	NA	NA
Balarabe (22)	F	8	not done	not done	16	pyridostigmine	remain stable	AM	visual impairment,numbness and paraesthesia of lower limbs, urinary and fecal incontinence	NA	2	methylprednisolone, physiotherapy	NA	NA	P	P	NA
O’Riordan (23)	F	NA (preceded NMOSD)	NA	NA	41	NA	well-controlled	ON	acute vision loss in the left eye	1	2	steroids CTX	NA	moderate paraparesis	NA	NA	SLE,ANA, anti-dsDNA Ab
Kimura (24)	F	47	48	hyperplasia	48	steroid, thymectomy	NA	AM	NA	1	3	steroid tacrolimus	steroid tacrolimus	evolved to recurrent myelitis	P	P	NA
Kimura (24)	M	48	50	normal	61	steroid, tacrolimus	worsening of MG was seen three times	ON	NA	1	1	NA	steroid tacrolimus	evolved to recurrent ON and myelitis	P	P	NA
Bibic (7)	F	36	not done	not done	40	PE,IVMP,RTX, IVIG	passed away in ICU	ON	NA	1	0	IVMP	AZA,HCQ, metacortandracin	passed away in ICU	N	P	hypothyroidism SLE anti-MuSK Ab
Bibic (7)	F	32	not done	not done	36	pyridostigmine	well-controlled	NMOSD	leg weakness, numbness, and pain in the lower extremities bilaterally	1	1	IVMP,PE,RTX,CTX	preventative immunotherapy was not initiated as NMOSD had been quiescent	quiescent	P	N	NA
Bates (25)	F	39	49	NA	54	pyridostigmine, thymectomy	well-controlled	NMOSD	right sided weakness	NA	NA	IVMP	eculizumab	improvement of strength nearly to baseline	P	P	anti-MOG Ab 1:40
Gotkine (26)	F	23	NA	NA	41	pyridostigmine, steroids,AZA, thymectomy	clinical remission	AM	paresthesia	0	2	NA	steroid	NA	P	NA	NA
Gotkine (26)	F	10	12	NA	26	pyridostigmine, steroids,AZA, thymectomy	full remission	AM	neck pain, upper and lower limb weakness, and urinary urgency	0	3	IVMP,PE	NA	remained wheelchair bound	P	P	ANA
Gotkine (26)	F	14	NA	NA	23	steroid, thymectomy	clinical remission	AM	acute bilateral leg weakness and urinary retention	0	1	NA	steroid	bilateral leg weakness and urinary retention improved	P	NA	NA
Tola (27)	F	27	37	hyperplasia	58	anticholinesterase thymectomy	well-controlled	ON	NA	2	4	IVMP,PE,	NA	symptoms partially improved	P	NA	dsDNA1:180, ANA1:360, SLE
PIñAR (28)	F	NA	not done	not done	49	prednison, pyridostigmine bromide	well-controlled	AM	weakness, hypoesthesia in the right leg back pain	NA	1	IVMP	prednison MMF	symptoms partially improved, but dysuria persisted	P	P(6.99)	NA
PIñAR (28)	F	NA	NA	NA	76	pyridostigmine	well-controlled	NMOSD	paresthesia weakness and urinary retention	NA	2	IVMP	prednison MMF RTX	symptoms partially improved, and mild paresis persisted	P	P	ANA,ENA, SS,hypothyroidism
Jarius (19)	F	22	not done	not done	20	NA	NA	ON	NA	6	5	NA	NA	NA	P	P	Hashimoto's thyroditis; TPOAb, TG-Ab
Kimura(24)	F	32	NA	NA	31	thymectomy	NA	AM	NA	0	7	IFN β	steroid, tacrolimus;	NA	NA	P	NA
Etemadifar (29)	F	42	not done	not done	33	pyridostigmine, AZA;	NA	ON	NA	3	1	PE mitoxantrone	prednisolone AZA	NA	P	P	NA
Yau (30)	F	56	not done	not done	51	pyridostigmine	well-controlled	ON	visual impairment in right eye	4	0	IVMP	AZA	no relapse of ON	P (8.83)	P	ANA
Bonner (31)	F	44 (3months since NMOSD diagnosis)	not done	not done	44	pyridostigmine	complete remission	AM	headache followed by imbalance, right sided extremity numbness and weakness	0	1	IVMP RTX	MMF	no relapse of NMOSD	P (0.53 )	P	N-methyl-D Aspartate receptor encephalitis,
Antoine (32)	M	49	49	thymoma	49	thymectomy, IVIG,PE	complete remission	NMOSD	dmyalgia, weakness, and blind ultimately	1	1	IVMP,PE, CTX	NA	able to walk using a cane blind	P	NA	NA
Furukawa (12)	F	63	not done	not done	63	prednisolone	remission	NMOSD	impairment of right visual, gait disturbance	1	1	IVMP	NA	neurological symptoms improved	P (141 )	N	ANA,SLE, Grave’s disease, autoimmune thyroid disease
Our patient	F	45	not done	not done	55	traditional Chinese medicine	well-controlled	NMOSD	dizziness, nausea, vomiting, gait disturbance	2	0	IVMP	prednison MMF inebilizumab	no obvious improvement in vision	P	P	Hashimoto's thyroditis

MG, myasthenia gravis; NMOSD, neuromyelits optica spectrum disorders; AChR, acetylcholine receptor; ON, optic neuritis; AM, acute myelitis; P, Positive; N, Negative; NA, not available; F, female; M, male; AZA, azathioprine; HCQ, hydroxychloroquine; PE, plasma exchange; CTX, cytoxan; ANA, antinuclear antibody; SLE, systemic lupus erythematosus; SS, Sjögren’s syndrome; IVMP, intravenous methylprednisolone pulse; MMF, mycophenolate mofetil.

In 50 patients with treatment details, 21 patients started with ON, 16 with myelitis, and 13 were diagnosed with NMOSD in the beginning. Additionally, of 44 patients with clear records of disease recurrence, 31 patients (70.5%) experienced frequent relapses of ON and myelitis, ranging from 1 to 15 attacks, averaging five. Twenty-three patients (63.9%) were treated with intravenous methylprednisolone (IVMP), 6 (16.7%) with plasma exchange (PE), and one (2.7%) with intravenous immunoglobulin (IVIG) during NMOSD acute phases. Regarding the treatment during remission phases, 10 patients (37.0%)were maintained with steroid or prednisolone alone, 14 patients(51.9%) had additional immunosuppressive agents, and 3 patients (11.1%) were supplemented with biological agents, including our patient. One patient passed away in ICU despite IVMP treatment, and 8 patients (28.6%) experienced persistent severe disabilities. Ten patients (35.7%) received completely symptom improvement, and 7 patients (25%) received partially symptoms improvement. Furthermore, most cases responded well to MG therapy, and the prognosis is often favorable. In our review, 13 out of 29 patients (44.8%) exhibited well-controlled symptoms or remained stable, while clinical remission or complete remission was reported in 11 patients (37.9%). However, 3 patients experienced MG crises. Therefore, for individuals with concurrent NMOSD and MG, it is essential to pay attention to the management of MG alongside NMOSD treatment.

## Discussion

Our patient was diagnosed with both AQP4 antibody-positive NMOSD and AChR antibody-positive MG. She initially presented with dizziness, nausea, vomiting, gait disturbance, and then vision loss. However, despite receiving standard treatment, her vision improved only slightly. In contrast, her MG symptoms, such as ptosis and dysarthria, were well-controlled prior to the NMOSD onset, with a QMGS score of 0. She had been treated with inebilizumab for one year follow-up, with no relapses reported to date.

Although the mechanisms underlying co-occurrence of NMOSD and MG remain unclear, it is hypothesized that the two diseases share common immune pathological mechanisms. In NMOSD, B cells contribute to pathogenesis through the production of pathogenic AQP4-IgG antibodies by plasmablasts (PBs) and plasma cells (PCs) (33), secretion of pro-inflammatory cytokines, and antigen presentation that activates autoreactive T cells (34). MG is due to the action of pathogenic antibodies secreted by PCs at the neuromuscular junction, leading to neuromuscular dysfunction. Both diseases have highly specific autoantibodies, which secreted by PBs and PCs differentiated from B cells. Moreover, genetic predispositions also contribute to the co-occurrence of NMOSD and MG. Human Leukocyte Antigen (HLA) -DPB1*05:01 in both southern Han Chinese and Japanese populations are linked to an increased risk of developing NMOSD (35). HLA-C07:01:01 is a well-characterized risk factor for MG. Additionally, HLA-DRB1*03:01 and HLA-DRB1*15:01 have been emerged as an independent risk allele for both disease (36). Certain HLA types have been pinpointed that correlate with the susceptibility to developing NMOSD and MG (37). When appropriate, HLA genotyping should be considered.

In patients with both conditions, MG typically precedes NMOSD and is often associated with thymectomy. A previous study reported that more than 50% of MG patients have their thymus removed (38). Our literature review showed that approximately 70% patients with both NMOSD and MG had undergone thymectomy. It is a higher proportion than in MG patients without NMOSD. However, it has been reported that NMOSD can develop in MG patients without thymectomy (8), as observed in our case. This indicates that thymectomy is not a necessary factor for the occurrence of NMOSD. One possible explanation is that AQP4, expressed at the peripheral neuromuscular junction, may act as a shared target for both diseases (39). The degeneration of the postsynaptic membrane induced by AChR antibodies may trigger AQP4 sensitization within the inflammatory environment of MG, consequently leading to autoimmunity against AQP4 (7). This explains why the MG patients without thymectomy would develop NMOSD. Therefore, thymectomy is not prerequisite for NMOSD onset in MG patients. In contrast, for patients who develop NMOSD after thymectomy, the expression of AQP4 in the thymus gland may play a role. The abnormal thymus associated with MG could generate anti-AQP4 antibodies (6). In some cases, an immune response against AQP4 on thymoma cells may trigger NMOSD. And there is another viewpoint that regulatory T cells in the adult thymus play a role in preventing the emergence of autoimmune diseases by keeping autoreactive cells in check. A reduction in regulatory T cells after thymectomy may contribute to the development of NMOSD (40).

In the literature review, many patients were found to have concurrent autoimmune conditions or other immune-related antibodies, such as ANA and dsDNA. Evidence suggests that over 25% of patients with autoimmune disorders are likely to develop another autoimmune condition, which can be either organ-specific or systemic-specific (40). Common co-occurring conditions include thyroid disease, SLE, SS, rheumatoid arthritis, antiphospholipid syndrome, ulcerative colitis and sarcoidosis, et al. Therefore, after the diagnosis of an autoimmune disease, screening for antibodies related to other autoimmune disorders is recommended. A similar situation is observed in patients with both NMOSD and MG. In most cases, MG precedes NMOSD by more than 10 years. Additionally, AQP4-Abs have been detected in some MG patients years even in the absence of clinical manifestations of NMOSD (8). Based on this, we recommend routine evaluation of AQP4-Abs in MG patients, as well as thyroid antibodies, ANA, and dsDNA et al. Moreover, clinical symptoms and signs are essential for determining whether MG coexists with NMOSD or only antibodies are present without active disease. It is also recognized that NMOSD can occur in MG patients even in the absence of AQP4-Abs. This highlights the importance of screening for NMOSD in MG patients, especially when clinical features overlap, regardless of antibody status. A thorough clinical evaluation and the use of advanced diagnostic techniques are crucial for accurately identifying coexisting autoimmune conditions.

The treatment strategies for NMOSD and MG have advanced rapidly in recent years, with novel therapeutic biologics targeting diverse mechanisms emerging. For NMOSD, three biologics have been approved by the U.S. Food and Drug Administration (FDA) for patients with NMOSD: eculizumab, satralizumab, and inebilizumab (41–43). Furthermore, rituximab (RTX) has class I evidence supporting its use in AQP4-IgG positive NMOSD (44). For MG, eculizumab is FDA-approved for refractory cases with efficacy supported by phase III trial data. Efgartigimod has also been approved for the treatment of generalized MG (45). Interleukin-6 inhibitors like satralizumab are under active evaluation (46). RTX has demonstrated promising results in MG, with studies reporting reductions in autoantibody levels and improvements in clinical symptoms (47).

MG tends to be a milder condition in patients with comorbid NMOSD and MG, and its relapse was rare once NMOSD developed. NMOSD appears to be more aggressive and tends to have recurrent attacks. In the case of our patient, MG remained stable, so a special focus on the treatment of NMOSD. NMOSD and MG are both B cell-mediated autoimmune diseases, making B cell-depleting therapies a logical therapeutic approach. Inebilizumab is FDA-approved for NMOSD. And evidence supports inebilizumab is effective for MG and may even outperform other anti-CD20 therapies such as ocrelizumab, ofatumumab, and obinutuzumab in this context (48). Additionally, inebilizumab has demonstrated potential in other humoral immune-mediated autoimmune diseases. Studies have indicated that it reduces the risk of flares in IgG4-related disease and increases the likelihood of achieving flare-free complete remission within one year (49). Phase I clinical trials of inebilizumab for the treatment of multiple sclerosis (NCT01585766) and systemic sclerosis (NCT00946699) have been completed, showing signals of clinical effectiveness (50). Phase III clinical trials for the treatment of systemic sclerosis (NCT05198557) and N-methyl-D-aspartate receptor encephalitis (NCT04372615)are currently underway (51).

It is important to acknowledge the limitations of our study to provide a balanced perspective on the findings. Firstly, the duration of the follow-up period was relatively short, limited to one year, which restricts conclusions about the long-term sustainability of the observed effects. Secondly, other than monitoring the quantity and functionality of immune cells such as T cells and B cells, glial fibrillary acidic protein(GFAP), neurofilament light chain(NfL) and AQP4 antibody level is crucial for comprehensively evaluating treatment efficacy and predicting prognosis. In light of these limitations, we encourage future research to address these gaps through studies with extended follow-up periods and more robust assessments in patients with NMOSD and MG.

## Data Availability

The original contributions presented in the study are included in the article/supplementary material. Further inquiries can be directed to the corresponding author.

## References

[1] Chatterton S Parratt JDE Ng K . Eculizumab for acute relapse of neuromyelitis optica spectrum disorder: Case report. Front Neurol. (2022) 13:951423. doi: 10.3389/fneur.2022.951423 36003301 PMC9393544

[2] Uzawa A Mori M Iwai Y Kobayashi M Hayakawa S Kawaguchi N . Association of anti-aquaporin-4 antibody-positive neuromyelitis optica with myasthenia gravis. J Neurol Sci. (2009) 287:105–7. doi: 10.1016/j.jns.2009.08.040 19729173

[3] Luzanova E Stepanova S Nadtochiy N Kryukova E Karpova M . Cross-syndrome: myasthenia gravis and the demyelinating diseases of the central nervous system combination. Systematic literature review and case reports. Acta Neurol Belg. (2023) 123:367–74. doi: 10.1007/s13760-022-01926-z 35699899

[4] Bagherieh S Afshari-Safavi A Vaheb S Kinai M Ghaffary EM Barzegar M . Worldwide prevalence of neuromyelitis optica spectrum disorder (NMOSD) and neuromyelitis optica (NMO): a systematic review and meta-analysis. Neurol Sci. (2023) 44:1905–15. doi: 10.1007/s10072-023-06617-y 36745300

[5] Bong JB Lee MA Kang HG . Newly diagnosed multiple sclerosis in a patient with ocular myasthenia gravis: A case report. Med (Baltimore). (2022) 101:e28887. doi: 10.1097/MD.0000000000028887 PMC8878705 35212290

[6] Zhu Y Wang B Hao Y Zhu R . Clinical features of myasthenia gravis with neurological and systemic autoimmune diseases. Front Immunol. (2023) 14:1223322. doi: 10.3389/fimmu.2023.1223322 37781409 PMC10538566

[7] Bibic VC Brust TB Burton JM . Neuromyelitis optica spectrum disorder presenting with concurrent autoimmune diseases. Mult Scler Relat Disord. (2019) 28:125–8. doi: 10.1016/j.msard.2018.12.028 30593981

[8] Leite MI Coutinho E Lana-Peixoto M Apostolos S Waters P Sato D . Myasthenia gravis and neuromyelitis optica spectrum disorder: a multicenter study of 16 patients. NEUROLOGY. (2012) 78:1601–7. doi: 10.1212/WNL.0b013e31825644ff PMC3348852 22551731

[9] Vaknin-Dembinsky A Abramsky O Petrou P Ben-Hur T Gotkine M Brill L . Myasthenia gravis-associated neuromyelitis optica-like disease: an immunological link between the central nervous system and muscle? Arch Neurol. (2011) 68:1557–61. doi: 10.1001/archneurol.2011.200 21825214

[10] Isbister CM Mackenzie PJ Anderson D Wade NK Oger J . Co-occurrence of multiple sclerosis and myasthenia gravis in british columbia. Mult Scler (2003) 9:550–3. doi: 10.1191/1352458503ms964oa 14664466

[11] Kay CS Scola RH Lorenzoni PJ Jarius S Arruda WO Werneck LC . NMO-IgG positive neuromyelitis optica in a patient with myasthenia gravis but no thymectomy. J Neurol Sci (2008) 275:148–50. doi: 10.1016/j.jns.2008.06.038 18703206

[12] Furukawa Y Yoshikawa H Yachie A Yamada M . Neuromyelitis optica associated with myasthenia gravis: characteristic phenotype in japanese population. Eur J Neurol (2006) 13:655–8. doi: 10.1111/j.1468-1331.2006.01392.x 16796591

[13] Kister I Gulati S Boz C Bergamaschi R Piccolo G Oger J . Neuromyelitis optica in patients with myasthenia gravis who underwent thymectomy. Arch Neurol (2006) 63:851–6. doi: 10.1001/archneur.63.6.851 16769866

[14] Nakamura M Nakashima I Sato S Miyazawa I Fujihara K Itoyama Y . Clinical and laboratory features of neuromyelitis optica with oligoclonal IgG bands. Mult Scler (2007) 13:332–5. doi: 10.1177/13524585070130030201 17439901

[15] Bichuetti DB Barros TM Oliveira EM Annes M Gabbai AA . Demyelinating disease in patients with myasthenia gravis. Arq Neuro. (2008) 66:5–7. doi: 10.1590/s0004-282x2008000100002 18392404

[16] Kohsaka M Tanaka M Tahara M Araki Y Mori S Konishi T . [A case of subacute myelitis with anti-aquaporin 4 antibody after thymectomy for myasthenia gravis: review of autoimmune diseases after thymectomy]. Rinsho Shinkeigaku. (2010) 50:111–3. doi: 10.5692/clinicalneurol.50.111 20196494

[17] Ogaki K Hirayama T Chijiiwa K Fukae J Furuya T Noda K . Anti-aquaporin-4 antibody-positive definite neuromyelitis optica in a patient with thymectomy for myasthenia gravis. Neurologist. (2012) 18:76–9. doi: 10.1097/NRL.0b013e318247bc91 22367834

[18] Hironishi M Ishimoto S Sawanishi T Miwa H Kawachi I Kondo T . [Neuromyelitis optica following thymectomy with severe spinal cord atrophy after frequent relapses for 30 years]. Brain Nerve. (2012) 64:951–5. doi: 10.11477/mf.1416101273 22868887

[19] Jarius S Paul F Franciotta D de Seze J Münch C Salvetti M . Neuromyelitis optica spectrum disorders in patients with myasthenia gravis: ten new aquaporin-4 antibody positive cases and a review of the literature. Mult Scler (2012) 18:1135–43. doi: 10.1177/1352458511431728 22183934

[20] Spillane J Christofi G Sidle KC Kullmann DM Howard RS . Myasthenia gravis and neuromyelitis opica: A causal link. Mult Scler Relat Disord (2013) 2:233–7. doi: 10.1016/j.msard.2013.01.003 25877729

[21] Ikeguchi R Shimizu Y Suzuki S Shimizu S Kabasawa C Hashimoto S . Japanese cases of neuromyelitis optica spectrum disorder associated with myasthenia gravis and a review of the literature. Clin Neurol Neurosurg (2014) 125:217–21. doi: 10.1016/j.clineuro.2014.07.036 25178916

[22] Balarabe SA Adamu MD Watila MM Jiya N . Neuromyelitis optica and myasthenia gravis in a young nigerian girl. BMJ Case Rep (2015). doi: 10.1136/bcr-2014-207362 PMC4567747 26338241

[23] O'Riordan JI Gallagher HL Thompson AJ Howard RS Kingsley DP Thompson EJ . Clinical, CSF, and MRI findings in devic's neuromyelitis optica. J Neurol Neurosurg Psychiatry (1996) 60:382–7. doi: 10.1136/jnnp.60.4.382 PMC1073888 8774400

[24] Kimura K Okada Y Fujii C Komatsu K Takahashi R Matsumoto S . Clinical characteristics of autoimmune disorders in the central nervous system associated with myasthenia gravis. J Neurol (2019) 266:2743–51. doi: 10.1007/s00415-019-09461-3 31342158

[25] Bates M Chisholm J Miller E Avasarala J Guduru Z . Anti-MOG and anti-AQP4 positive neuromyelitis optica spectrum disorder in a patient with myasthenia gravis. Mult Scler Relat Disord (2020) 44:102205. doi: 10.1016/j.msard.2020.102205 32526697

[26] Gotkine M Fellig Y Abramsky O . Occurrence of CNS demyelinating disease in patients with myasthenia gravis. Neurology. (2006) 67:881–3. doi: 10.1212/01.wnl.0000234142.41728.a0 16966558

[27] Tola MR Casetta I Granieri E Caniatti LM Monetti VC Pascarella R . Systemic lupus erythematosus related recurrent transverse myelitis in a patient with myasthenia gravis and multiple sclerosis. Eur Neurol (1996) 36:327–8. doi: 10.1159/000117285 8864720

[28] Piñar Morales R Todorova Petrova M Barrero Hernández FJ . Copresence of myasthenia gravis and neuromyelitis optica: 2 case reports. Neurologia (2021) 36:174–6. doi: 10.1016/j.nrl.2020.02.008 32565034

[29] Etemadifar M Abtahi SH Dehghani A Abtahi MA Akbari M Tabrizi N . Myasthenia gravis during the course of neuromyelitis optica. Case Rep Neurol (2011) 3:268–73. doi: 10.1159/000334128 PMC3224523 22125527

[30] Yau GS Lee JW Chan TT Yuen CY . Neuromyelitis optica spectrum disorder in a chinese woman with ocular myasthenia gravis: First reported case in the chinese population. Neuroophthalmology. (2014) 38:140–4. doi: 10.3109/01658107.2013.879903 PMC5123045 27928290

[31] Bonner K Aboul Nour H Memon AB . Overlapping autoimmune neurological syndrome: A case report of triple-positive antibody. Cureus. (2022) 14:e29379. doi: 10.7759/cureus.29379 36168655 PMC9505631

[32] Antoine JC Camdessanché JP Absi L Lassablière F Féasson L . Devic disease and thymoma with anti-central nervous system and antithymus antibodies. Neurology. (2004) 62:978–80. doi: 10.1212/01.wnl.0000115168.73299.88 15037705

[33] Jasiak-Zatonska M Kalinowska-Lyszczarz A Michalak S Kozubski W . The immunology of neuromyelitis optica-current knowledge, clinical implications, controversies and future perspectives. Int J Mol Sci. (2016) 17:273. doi: 10.3390/ijms17030273 26950113 PMC4813137

[34] Bennett JL Aktas O Rees WA Smith MA Gunsior M Li Y . Association between B-cell depletion and attack risk in neuromyelitis optica spectrum disorder: An exploratory analysis from N-MOmentum, a double-blind, randomised, placebo-controlled, multicentre phase 2/3 trial. EBioMedicine. (2022) 86:104321. doi: 10.1016/j.ebiom.2022.104321 36370634 PMC9664402

[35] Jarius S Aktas O Ayzenberg I Bellmann-Strobl J Berthele A Giglhuber K . Update on the diagnosis and treatment of neuromyelits optica spectrum disorders (NMOSD) - revised recommendations of the Neuromyelitis Optica Study Group (NEMOS). Part I: Diagnosis and differential diagnosis. J Neurol. (2023) 270:3341–68. doi: 10.1007/s00415-023-11634-0 PMC10267280 37022481

[36] Vakrakou A Chatzistamatiou T Koros C Karathanasis D Tentolouris-Piperas V Tzanetakos D . HLA-genotyping by next-generation-sequencing reveals shared and unique HLA alleles in two patients with coexisting neuromyelitis optica spectrum disorder and thymectomized myasthenia gravis: Immunological implications for mutual aetiopathogenesis? Mult Scler Relat Disord. (2022) 63:103858. doi: 10.1016/j.msard.2022.103858 35594634

[37] Saruhan-Direskeneli G Hughes T Yilmaz V Durmus H Adler A Alahgholi-Hajibehzad M . Genetic heterogeneity within the HLA region in three distinct clinical subgroups of myasthenia gravis. Clin Immunol. (2016) 166-167:81–8. doi: 10.1016/j.clim.2016.05.003 27181991

[38] Taioli E Paschal PK Liu B Kaufman AJ Flores RM . Comparison of conservative treatment and thymectomy on myasthenia gravis outcome. Ann Thorac Surg. (2016) 102:1805–13. doi: 10.1016/j.athoracsur.2016.08.052 28148454

[39] Wakayama Y . Aquaporin expression in normal and pathological skeletal muscles: a brief review with focus on AQP4. J BioMed Biotechnol. (2010) 2010:731569. doi: 10.1155/2010/731569 20339523 PMC2842974

[40] Castro-Suarez S Guevara-Silva E Caparó-Zamalloa C Cortez J Meza-Vega M . Neuromyelitis optica in patients with myasthenia gravis: Two case-reports. Mult Scler Relat Disord. (2020) 43:102173. doi: 10.1016/j.msard.2020.102173 32442888

[41] Frampton JE . Inebilizumab: first approval. Drugs. (2020) 80:1259–64. doi: 10.1007/s40265-020-01370-4 PMC7387876 32729016

[42] Pittock SJ Berthele A Fujihara K Kim HJ Levy M Palace J . Eculizumab in aquaporin-4-positive neuromyelitis optica spectrum disorder. N Engl J Med. (2019) 381:614–25. doi: 10.1056/NEJMoa1900866 31050279

[43] Yamamura T Kleiter I Fujihara K Palace J Greenberg B Zakrzewska-Pniewska B . Trial of satralizumab in neuromyelitis optica spectrum disorder. N Engl J Med. (2019) 381:2114–24. doi: 10.1056/NEJMoa1901747 31774956

[44] Redenbaugh V Flanagan EP . Monoclonal antibody therapies beyond complement for NMOSD and MOGAD. Neurotherapeutics. (2022) 19:808–22. doi: 10.1007/s13311-022-01206-x PMC9294102 35267170

[45] Heo YA . Efgartigimod: first approval. Drugs. (2022) 82:341–8. doi: 10.1007/s40265-022-01678-3 PMC8855644 35179720

[46] Menon D Bril V . Pharmacotherapy of generalized myasthenia gravis with special emphasis on newer biologicals. Drugs. (2022) 82:865–87. doi: 10.1007/s40265-022-01726-y PMC9152838 35639288

[47] Carnero Contentti E Correale J . Neuromyelitis optica spectrum disorders: from pathophysiology to therapeutic strategies. J Neuroinflamm. (2021) 18:208. doi: 10.1186/s12974-021-02249-1 PMC8444436 34530847

[48] Furman MJ Meuth SG Albrecht P Dietrich M Blum H Mares J . B cell targeted therapies in inflammatory autoimmune disease of the central nervous system. Front Immunol. (2023) 14:1129906. doi: 10.3389/fimmu.2023.1129906 36969208 PMC10034856

[49] Stone JH Khosroshahi A Zhang W Della Torre E Okazaki K Tanaka Y . Inebilizumab for treatment of igG4-related disease. N Engl J Med. (2024) NEJMoa2409712. doi: 10.1056/NEJMoa2409712 39541094

[50] Chen D Gallagher S Monson NL . Inebilizumab, a B cell-depleting anti-CD19 antibody for the treatment of autoimmune neurological diseases: insights from preclinical studies. J Clin Med. (2016) 5:107. doi: 10.3390/jcm5120107 27886126 PMC5184780

[51] Hara M Nakajima H . Anti-NMDAR encephalitis with poor recovery on steroid pulse and IVIg: practical approach to intensive immunotherapy. Brain Nerve. (2022) 74:433–42. doi: 10.11477/mf.1416202061 35589628

